# Supplementary material for: Multi‐dataset identification of innovative feature genes and molecular mechanisms in keratoconus
Source: J Cell Mol Med. 2024 Sep 19;28(18):e70079. doi: 10.1111/jcmm.70079 (PMC11412914; doi:10.1111/jcmm.70079)
Supplement: Supplementary file 2 — Material S2. [file JCMM-28-e70079-s002.zip › 7.GSEA analysis/6.SNORA74B/KEGG_O_GLYCAN_BIOSYNTHESIS.html]

Details for gene set KEGG\_O\_GLYCAN\_BIOSYNTHESIS[GSEA]

|  || Dataset | SNORA74B.group.cls#h\_versus\_l |
| Phenotype | group.cls#h\_versus\_l |
| Upregulated in class | l |
| GeneSet | KEGG\_O\_GLYCAN\_BIOSYNTHESIS |
| Enrichment Score (ES) | -0.47841293 |
| Normalized Enrichment Score (NES) | -1.3664949 |
| Nominal p-value | 0.13060428 |
| FDR q-value | 0.34430024 |
| FWER p-Value | 0.92 |
Table: GSEA Results Summary

  

Fig 1: Enrichment plot: KEGG\_O\_GLYCAN\_BIOSYNTHESIS      
 Profile of the Running ES Score & Positions of GeneSet Members on the Rank Ordered List

  

| SYMBOL | TITLE | RANK IN GENE LIST | RANK METRIC SCORE | RUNNING ES | CORE ENRICHMENT || 1 | GALNT8 | na | 3796 | 0.267 | -0.0566 | No |
| 2 | GALNTL6 | na | 4802 | 0.232 | -0.0398 | No |
| 3 | ST6GALNAC1 | na | 5069 | 0.223 | -0.0034 | No |
| 4 | B3GNT6 | na | 6035 | 0.191 | 0.0065 | No |
| 5 | GALNT5 | na | 6934 | 0.164 | 0.0130 | No |
| 6 | GALNT12 | na | 8717 | 0.120 | -0.0146 | No |
| 7 | GALNT2 | na | 8917 | 0.115 | 0.0024 | No |
| 8 | GCNT4 | na | 12659 | 0.051 | -0.0953 | No |
| 9 | ST3GAL1 | na | 13468 | 0.042 | -0.1103 | No |
| 10 | GALNT1 | na | 14182 | 0.034 | -0.1242 | No |
| 11 | GALNT9 | na | 14772 | 0.027 | -0.1359 | No |
| 12 | B4GALT5 | na | 14912 | 0.025 | -0.1349 | No |
| 13 | C1GALT1C1 | na | 15344 | 0.021 | -0.1431 | No |
| 14 | GALNT3 | na | 15590 | 0.019 | -0.1465 | No |
| 15 | GALNT10 | na | 16605 | 0.008 | -0.1741 | No |
| 16 | C1GALT1 | na | 17956 | -0.006 | -0.2119 | No |
| 17 | GALNT7 | na | 18331 | -0.010 | -0.2207 | No |
| 18 | GALNT18 | na | 22036 | -0.052 | -0.3172 | No |
| 19 | GALNT11 | na | 23688 | -0.076 | -0.3497 | No |
| 20 | GALNT4 | na | 24761 | -0.094 | -0.3621 | No |
| 21 | GALNTL5 | na | 24822 | -0.095 | -0.3450 | No |
| 22 | GALNT6 | na | 25226 | -0.103 | -0.3364 | No |
| 23 | GALNT15 | na | 29487 | -0.209 | -0.4178 | No |
| 24 | ST3GAL2 | na | 31591 | -0.300 | -0.4192 | Yes |
| 25 | GCNT3 | na | 32490 | -0.354 | -0.3752 | Yes |
| 26 | GALNT16 | na | 33199 | -0.413 | -0.3140 | Yes |
| 27 | GALNT13 | na | 33772 | -0.489 | -0.2339 | Yes |
| 28 | GCNT1 | na | 33888 | -0.510 | -0.1365 | Yes |
| 29 | GALNT14 | na | 34615 | -0.813 | 0.0033 | Yes |
Table: GSEA details [plain text format]

  

Fig 2: KEGG\_O\_GLYCAN\_BIOSYNTHESIS      
 Blue-Pink O' Gram in the Space of the Analyzed GeneSet

  

Fig 3: KEGG\_O\_GLYCAN\_BIOSYNTHESIS: Random ES distribution      
 Gene set null distribution of ES for **KEGG\_O\_GLYCAN\_BIOSYNTHESIS**
